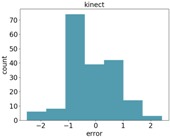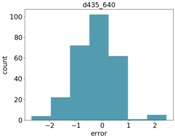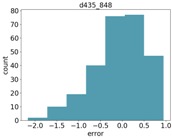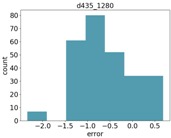# Comparing RGB-D Sensors for Close Range Outdoor Agricultural Phenotyping

**DOI:** 10.3390/s18124413

**Published:** 2018-12-13

**Authors:** Adar Vit, Guy Shani

**Affiliations:** Software and Information Systems Engineering, Ben Gurion University, Beer Sheva 84105, Israel; shanigu@bgu.ac.il

**Keywords:** RGB-D sensors, empirical analysis, sensors in agriculture, phenotyping, Microsoft Kinect, Intel d-435, Intel sr300, Orbbec astra s

## Abstract

Phenotyping is the task of measuring plant attributes for analyzing the current state of the plant. In agriculture, phenotyping can be used to make decisions concerning the management of crops, such as the watering policy, or whether to spray for a certain pest. Currently, large scale phenotyping in fields is typically done using manual labor, which is a costly, low throughput process. Researchers often advocate the use of automated systems for phenotyping, relying on the use of sensors for making measurements. The recent rise of low cost, yet reasonably accurate, RGB-D sensors has opened the way for using these sensors in field phenotyping applications. In this paper, we investigate the applicability of four different RGB-D sensors for this task. We conduct an outdoor experiment, measuring plant attribute in various distances and light conditions. Our results show that modern RGB-D sensors, in particular, the Intel D435 sensor, provides a viable tool for close range phenotyping tasks in fields.

## 1. Introduction

The constant increase of the world’s population increases the needs for technological developments in the agriculture industry. In order to meet the growing demand for food, the agriculture industry has to develop technological tools which will allow us to increase crop production [1]. The process of crop phenotyping, including the extraction of visual traits from plants, allows farmers to examine their crops and infer important properties concerning the crop status, such as insufficient irrigation, or developing diseases [2].

Consequently, there is an urgent need for the development of novel methods in phenotyping for a non-destructive determination of diverse traits under field conditions [3]—non-destructive, meaning that phenotypic data can be collected from the same organism over the course of a long experiment. They are also amenable to automation, making it feasible to study large sample sizes for increased statistical power. The image-based phenotyping approach aims to perform extraction phenomics based on obtained images data in non-destructive way [4]. Recently, many new technologies use computer vision methods for phenotyping [5].

Field and greenhouse phenotyping—measuring plant phenotypes in standard growing conditions, rather than in a controlled lab, is a difficult challenge [6]. Field conditions are notoriously heterogeneous and the inability to control environmental factors, such as lighting and occlusion, makes results difficult to interpret; however, results from controlled environments are far removed from the situation plants will experience in the field and, therefore, are difficult to extrapolate to the field [2]. Furthermore, given the slim profit margins in agriculture, farmers cannot invest in costly sensors for phenotyping. Low-cost sensors can be used by the farmer, whether in a field or in a greenhouse, in order to allow for affordable phenotyping.

Indeed, many low cost visual sensors were developed in the last decade. For phenotyping, where one often needs to measure size, length, or width, RGB-D sensors are a viable alternative. RGB-D sensors provide, in addition to the three color channels (RGB – Red, Green, Blue), a depth channel (D), measuring the distance from the sensor to a point in the image. Using this information, we can estimate, e.g., the length and width of a stem, or the size of a fruit.

There are several alternative technologies on which low cost RGB-D sensors are based. Optical techniques for image acquisition can be divided into passive and active. Passive methods use the reflection of natural light on a given target to measure its shape. Active methods enhance shape acquisition by using an external lighting source that provides additional information [7]. For example, time-of-flight (TOF) sensors measure depth by estimating the time delay from light emission to light detection. Structured-light sensors combine the projection of a light pattern with a standard 2D camera and measure depth via triangulation [8]. Active stereoscopy sensors looks for artificially projected features from multiple cameras to reconstruct a 3D shape, using triangulation and epipolar geometry theory [7]. Our work focuses on active sensors.

In this paper, we evaluate four different low cost RGB-D sensors for agricultural phenotyping tasks. We compare the following sensors: Microsoft Kinect ii (Microsoft Corporation, Redmond, WA, USA), Orbbec Astra s (Orbbec 3D Tech. Intl. Inc, Shenzhen, China), Intel sr300 (Intel Corporation, Santa Clara, CA, USA), and Intel d435 (Intel Corporation, Santa Clara, CA, USA). We evaluate all sensors in identical outdoor settings, taking measurements of six corn plants, and two tomatoes of different colors and sizes. In addition, we have taken measurements of two plastic balls of different colors and sizes, as two reference objects of known diameter. Measurements were taken throughout the day in varying lighting conditions, and from varying distances, ranging from 20 cm to 150 cm.

We analyze the depth information received from the sensors to identify the fill rate—the portion of missing depth information over the objects of interest. The gathered images were used to train deep learning segmentation models for all object types (corn stalks, tomatoes, balls), which is an important task in phenotyping, and we report the quality of the resulting models. In addition, we have trained segmentation models using the depth information to estimate whether depth can enhance the quality of the models. We also use the depth information to estimate the known diameter of the plastic balls, and we compute the error of the various sensors.

Our results indicate that both the Microsoft Kinect ii and the Intel d435 provide much superior results to the Intel sr300 and the Orbbec Astra s in our settings. For many phenotyping applications, such as mounting sensors on drones [9,10], the lower weight and reduced energy demands of the Intel d435 make it an attractive choice for agricultural outdoor applications.

In addition, we present an application demonstrating the capabilities of the data collected by depth sensors, for estimating the width of maize stems in field condition from close range, using the Intel d435.

## 2. Related Work

We now review related work. We begin with reviewing some research on the use of RGB-D sensors in agricultural applications, and then discuss research on comparing RGB-D sensors, both in general applications and specifically in agricultural settings.

### 2.1. Using RGB-D in Agriculture Applications

In recent years, the use of RGB-D sensors has been increasing due to the ability of these sensors to simultaneously capture depth and color images of the scene. Efficiently mapping the depth and color data results in a colored point cloud in a 3D spatial domain [11], which is very useful in many applications.

Chéné et al. [12] showed the potential and ability of RGB-D imaging systems for 3D measurements in the context of plant phenotyping, in what appears to be the first published application with plants. They showed that Microsoft Kinect I can resolve individual leaves, allowing automated measurement of leaf orientation in indoor environments.

A wide variety of phenotyping tasks have been approached using RGB-D data. Xia et al. [13] presented an algorithm for leaf segmentation using RGB-D data. Using images of greenhouse paprika leaves, they demonstrate their ability to capture leaf measurements using an RGB-D camera. Ref. [14] estimated the size of sweet onions in indoor conditions, and Ref. [15] used the RGB-D camera for size estimation of mango fruits on trees in field conditions. Ref. [16] showed the ability to measure the canopy structure of small plants in the field. Azzari et al. [17] used the Microsoft Kinect I sensor to characterize the vegetation structure. Jiang et al. [18] presented an algorithm for accurately quantifying cotton canopy size in field conditions. They showed that the multidimensional traits and multivariate traits were better yield predictors than traditional univariate traits, confirming the advantage of using 3D imaging modalities. Sa et al. [19] presented 3D visual detection method for detecting peduncles of sweet peppers in the field using short-range depth camera Intel Real Sense F200 (Intel Corporation, Santa Clara, CA, USA).

Only a handful of studies use depth sensors for cereal phenotyping. Cereals are especially challenging, due to their relatively narrow stem, leaves, and head. Sodhi et al. [20] presented an automated method of mapping 2D images collected in an outdoor sorghum field and greenhouse to segmented 3D plant units that are of interest for phenotyping, using a multi-camera sensor.

### 2.2. Object Detection in Agriculture

Object detection is the task of identifying the positions of instances of semantic objects of a certain class in a given image using computer vision algorithms. Object detection in an image is a crucial aspect in the development of agricultural applications. In order to harvest fruits or vegetables, navigate in the field, or spray selectively, the object location in an image has to be determined. This allows us to position robotic arms accordingly, to identify obstacles, or to estimate the object’s characteristics such as ripeness or size [21].

Despite many years of research in agricultural oriented object detection, there are still many problems that hinder implementation of object detection in agricultural applications [22]. The highly variable and uncertain outdoor environment with changing illumination conditions, along with the complex plant structure and variable product shape and size make it hard to find a global solution to the detection of objects in the complex and unstructured agricultural environment [21]. Furthermore, in field conditions, there is a high probability of occlusion, where some plant parts, such as leaves, partially or fully hide the object of interest from the sensor. Handling occlusions is a major difficulty in phenotyping applications [23,24]. Depth information can help in identifying, if not resolving, occlusions, allowing an algorithm to detect the occluding objects that are closer to the sensor [13].

### 2.3. Comparing RGB-D Sensors

RGB-D sensors were compared in several different studies, using varying metrological methods to evaluate the performance of the sensors under certain conditions and environments. For example, Sabattini et al. [25] and Beltran and Basañez [26] compared the Microsoft Kinect I, which is based on structured-light technology, vs. the PointGrey Bumblebee2 Stereo–camera for localizing a mobile robot. The performance of the two cameras was compared using a mobile robot and 2D landmarks. Their work demonstrate experimentally that stereo cameras have a smaller error in the determination of the 3D position of known points in the image due to its better resolution.

Several studies [27,28,29], compared between two versions of Kinect: Microsoft Kinect I and Microsoft Kinect ii. Samir et al. [27] showed that the accuracy of v2 is slightly better than v1 with regard to the purpose of respiratory motion tracking, while Amon et al. [28] showed better performance for v2 in estimating the area of detection, as well as the rotation accuracy of two versions of the face tracking system of the Microsoft Kinect sensor. In [29], the comparison concentrated on depth image data. Their goal was to investigate the accuracy and precision of depth images of both devices. Accuracy is defined to be the difference or the offset of a measured depth value compared to a ground truth distance. Precision is defined as the repeatability of subsequent depth measurements under unchanged conditions. They investigate the influence of temperature, distance and object color on the captured depth images.

Diaz et al. [30] compare two generations of RGB-D sensors by evaluating the performances of Asus Xtion Pro (ASUSTeK Computer Inc., Taipei, Taiwan), a structured light based camera, and Microsoft Kinect ii. This evaluation considers the tasks of 3D reconstruction and object recognition. The quantitative comparison with respect to ground truth obtained using a metro-logical laser scanner revealed that Microsoft Kinect ii provides less error in the mapping between the RGB and depth frames, and the obtained depth values are more constant with distance variations.

Guidi et al. [31] compared between five low-cost 3D cameras with three different technologies by analyzing them in terms of systematic errors and random errors. They tested the cameras on a reference plain, made of a rectangular piece of float glass. Their tests have analyzed the range from 550 mm to 1450 mm, giving acceptable results with all the devices only between 550 mm and 1150 mm. Their results exhibit a global uncertainty similar for all the primesense-based devices, when the worst results are produced by the Realsense-based unit. They showed that the five low-cost 3D sensors they compared can certainly cater to gesture tracking and understanding.

The most profound and comprehensive was made in [7], they presented 20 3D cameras commercially available that use varying technologies—structured light, time of flight, and active and passive stereoscopy. They focused on indoor metrological evaluations on the state-of-the-art device in each technology: Microsoft Kinect ii (time of flight), Orbbec Astra s (structured light) and Intel d435 (active stereoscopy). They showed that the uncertainty in the depth measurement using a TOF camera scales linearly with the depth, hence providing reliable measurement at longer ranges. The Orbbec Astra s and the Intel RS400TM, on the other hand, which are based on the triangulation principle, provide a depth measurement in which the uncertainty grows quadratically. Hence, their usage is preferred for short-range applications.

Moreover, their work showed that the SR400TM generation of 3D camera provided by Intel proved to have outstanding performance when compared to other triangulation-based devices and for embedded applications, and that the RS400TM generation is a valuable device for shape acquisition. Although they used the same sensors as we do, we evaluate sensor performance in outdoor conditions and for agriculture phenotyping tasks.

Kazmi et al. [32] compared TOF cameras with stereo cameras for agricultural applications by evaluating close-range depth imaging of leaves indoor and outdoor, under shadow and sunlight conditions, by varying the exposure of the sensors. Their evaluation metrics focused on analyzing the depth data by aggregating it across several frames using various statistical measures. Their work concludes that TOF cameras are sensitive to ambient light, and stereo vision is relatively more robust for outdoor lighting.

Wang et al. [15] estimate mango fruit size on trees in field conditions. Three low-cost distance measurement technologies were compared for use in a fruit sizing system, to be mounted on a moving platform equipped with LED illumination for night imaging of mango orchards. For estimation of camera-to-fruit distance, low-cost examples of three distance measurement technologies were compared under lab conditions (fluorescent lighting): Zed (stereo vision camera) (San Francisco, CA, USA), Leica (TOF laser) (Weitzlal, Germany) and the Microsoft Kinect ii (TOF and RGB-D camera). The distance measurement was taken using three materials, varying in their level of diffuse reflectance, which were placed at 14 positions ranging from 0.56 to 5.4 m. In addition, the ceramic tile for Microsoft Kinect ii distance measurement was repeated outdoors at times from early afternoon to after sunset. The Zed stereo depth imaging technique was inferior to other technologies. In direct sunlight with a ceramic target, the Microsoft Kinect ii failed to measure a distance over 3.5 m.

## 3. Materials and Methods

We now report the experiment that was used to collect the data. In the experiment, four different RGB-D sensors were used in an outdoor scenario to take measurements of young corn plants and tomatoes.

### 3.1. RGB-D Sensors

**Figure 1:**
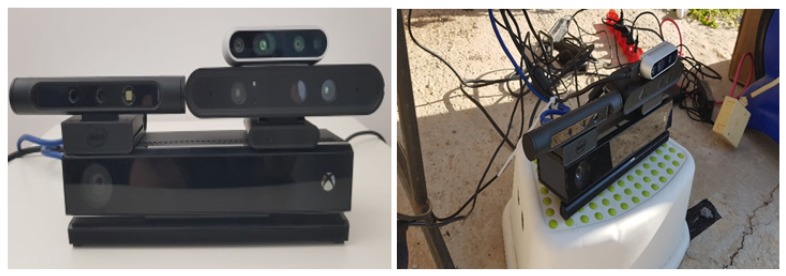
The setup of the four sensors used in the experiment. Kinect II at the bottom, the Astra Pro on the left, the SR300 on the right, and the gray D435 on top of the SR300.

We compare the following RGB-D sensors (Figure 1): Orbbec Astra s (https://orbbec3d.com/product-astra/), Microsoft Kinect ii (https://developer.microsoft.com/en-us/windows/kinect), Intel sr300 (https://software.intel.com/en-us/realsense/sr300), and Intel d435 (https://realsense.intel.com/stereo/). Table 1 shows the properties of all cameras. We now briefly review the sensors.

#### 3.1.1. Astra S

The Astra S sensor is manufactured by Orbbec company in Shenzhen, China. The camera is based on structured-light technology and it is designed for short-range measurements. The structured-light technology uses a single camera with a structured pattern projected on the scene. An infra-red (IR) projector projects a codified pattern embedding sufficient structure to provide unique correspondence. The direction of the structured pattern is known a priori, allowing triangulation based on the pattern [7]. The device contains an RGB sensor, IR sensors and a coded pattern projector. In addition, the device includes two microphones and an advanced eye protector.

#### 3.1.2. Microsoft Kinect II

The Microsoft Kinect ii sensor for Windows was released in 2014, along with a supporting SDK (Software Development Kit), allowing human body and face tracking. The device is based on Time-of-Flight (TOF) technology, estimating distance based on the known speed of light. Such sensors measure the time-of-flight of a light signal between the camera and the subject for each point of the image. The device requires a powerful illumination system with relatively high energy consumption. The device contains a full HD RGB camera which is registered with an IR camera. The device also includes IR emitters and a microphone.

#### 3.1.3. Intel©RealSense^TM^

Intel RealSense^TM^ provides an open platform for developers to incorporate Intel’s perceptual devices in their applications. The LibRealSense^TM^ cross-platform API (Application Programming Interface) provides several tools for managing the sensor’s streams, as well as advanced functions for background removal, hand tracking, fine face recognition and 3D scanning.

#### 3.1.4. Intel©SR300

Intel©SR300 was released in 2016 and it is the second generation of front-facing Intel©RealSense^TM^ cameras. Similar to the Astra sensor, the device is based on structured light technology and it is also designed for short ranges. The SR300 is a subassembly camera product that implements a short range (SR), coded light, and 3D imaging system. Along with an infra-red laser projector, the subassembly includes a Fast VGA (Video Graphics Array) infra-red camera and a 2-M pixel RGB color camera with an integrated image signal processor [33]. The cameras are factory-calibrated and the intrinsic and extrinsic parameters of the sensors are stored on board, easily accessible via the librealsense APIs.

#### 3.1.5. Intel©D435

Intel d435 is part of Intel’s D400^TM^ series, featuring the D435^TM^ and the D415^TM^. The Intel D435 depth camera is based on infra-red active stereoscopy technology, with a global shutter sensor. The depth is estimated in hardware through an imaging ASIC that processes the infra-red stream together with the RGB stream. The device performs frame correlation with a census cost function to identify homologous points and reconstructs the disparity. As it is based on active stereoscopy, an infra-red dot-pattern projector adds textures to the scene, to cope with low-texture environments, where the D435 has a randomly focused dot pattern. Both structured light and active stereoscopy are based on the same triangulation principle [7].

In our experiment, we tested the D435 with three modes: 1280 × 720 pixel resolution, 848 × 480 pixel resolution and 640 × 480 pixel resolution.

### 3.2. Measured Objects

To test the performance of the RGB-D sensors, we took measurements of six different young corn plants, organized in two rows of 3, to simulate the foreground-background setting in fields. The plants were five weeks old, and approximately 50 cm tall, and were planted in black plastic pots, allowing us to easily move them to different positions. The plants have not yet developed flowers or corn ears and husk.

In addition, we added two different types of tomatoes, allowing us to evaluate the usability of the captured images for object identification other than corn stems. We used one regular red tomato, and one oval orange cherry tomato, positioning them near the corn stalks.

In addition, for reliable measurements of object width, we added two plastic balls: a green ball with a diameter of 50 mm, and a smaller yellow ball with a diameter of 7.8 mm. The ball will be later used to evaluate the ability of the various RGB-D sensors for the important task of computing the width and length of an object of interest, such as stems, fruits, and leaves.

**Figure 2:**
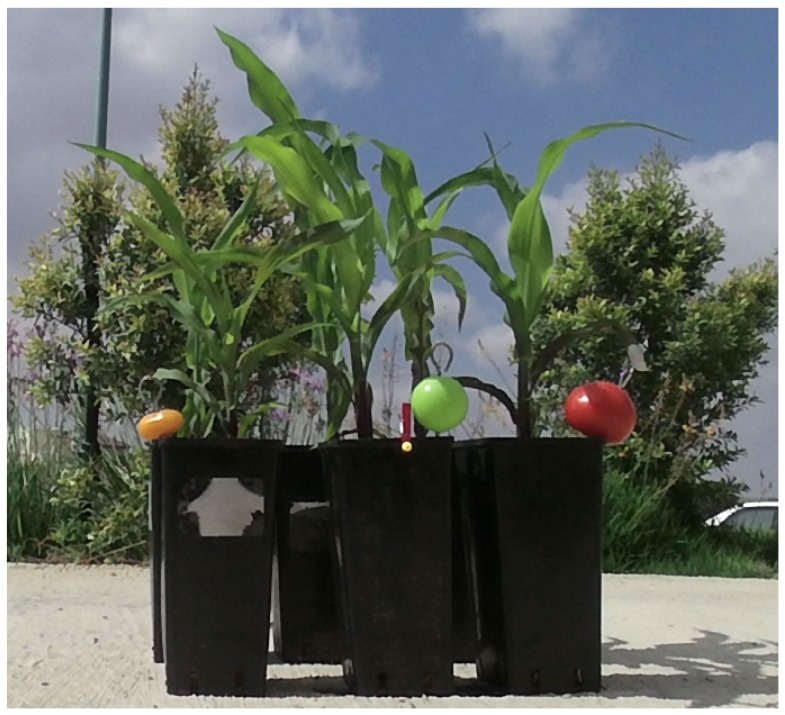
The experimental setup as captured by the Kinect sensor from 1 m. Six corn plants in black plastic pots arranged in two rows. Added tomatoes—a red tomato, an orange cherry tomato. Two plastic balls for size measurements—a larger green ball and a smaller yellow ball.

Figure 2 shows the setting of the corn plants, tomatoes, and plastic balls.

### 3.3. Procedure

In order to evaluate the performance of the RGB-D sensors for close range at various lighting conditions, we conducted an experiment in an outdoor environment. We assembled an imaging platform which employed a number of sensors: Orbbec Astra s, Microsoft Kinect ii, Intel sr300 and Intel d435. The Orbbec Astra s and the Intel d435 sensors were positioned next to each other, directly above the Microsoft Kinect ii. The Intel d435 sensor was positioned above the Orbbec Astra s. The sensors’ assembly was fixed during the entire experiment, and the sensors were not moved. Due to the similarities in the sensors’ technologies, some sensors cannot be used simultaneously on the same object. Specifically, the Intel sr300 and the Microsoft Kinect ii sensors caused mutual interference in the depth information. We used three different computers, allowing us to reduce the time between measurements of different sensors. For the Microsoft Kinect ii and the Orbbec Astra s, we used the official SDK for capturing RGB-D images, and the librealsense SDK was used for the Intel cameras.

As our goal is evaluation for short distances, our target objects were placed at seven positions ranging from 0.2 to 1.5 m. After measurements were taken, the pots were moved to the next position. The image acquisition process was repeated in twelve cycles, at various lighting conditions, from sunrise to sunset. At each cycle, we measured the luminous flux using the ambient light sensor of a Galaxy S8. Gutierrez-Martinez et al. [34] tested the accuracy of measurement of light by ambient light sensor in smartphones. Their study shows how the smartphones can be used in lighting measurement tasks when high precision of data is not required, being a great tool to have a reference of the luminance levels.

The lux values that the sensor provided were categorized into four ranges (http://stjarnhimlen.se/comp/radfaq.html):

1. Sunrise light: up to 1000 lux.
2. Overcast lighting: from 1000 to 10,000 lux.
3. Full daylight (vut not direct sun): from 10,000 to 32,000 lux.
4. Direct sunlight: 32,000 lux and above.

### 3.4. Fill Rate

It is often the case that some pixels in a depth image contain no depth information (typically marked as 0 depth), or contain incorrect depth measurements, such as distances which are much closer, or much farther, than the actual distance to the object of reference. The fill rate of a sensor is the portion of pixels that contain valid measurements within a region of interest (ROI). The fill rate is critical for tasks such as the segmentation of objects, or the measurement of width and length.

As we are interested in agricultural applications, our ROIs are the corn stems, the tomatoes, and the plastic balls. We calculate the fill rate of depth values in each ROI for each distance in each cycle. We consider a pixel measurement to be valid if it has a non-zero value, and it is within three standard deviations from the mean of non-zero depth values in the ROI.

### 3.5. Object Detection Using Deep-Learning

In order to compare the quality of the data produced by the sensors for the task of object detection, we use here a state-of-the-art algorithm, the Mask R-CNN model [35]. Our goal was to detect three different classes: the larger green ball, the tomato fruit, and the corn stem. In all of the experiments, we split the images by the time of day to train, validate, and test. There were 12 different time points where an image capture cycle for all distances had begun. Images from three cycles were used for testing, images from one cycle were used for validation, and images from the other eight cycles were used for training the models. As we are interested in comparing the images on identical algorithmic settings, we did not perform any hyper-parameter tuning, training all models with the same algorithmic configuration. Each model was trained for 60 epochs, where the last epoch contained also the validation set.

### 3.6. Object Size Estimation

Measuring the size of a fruit [15], or the width of a leaf or a stem [36] can be an important phenotype that can indicate the plant condition. We therefore examined the capabilities of the sensors to approximate the size of two different objects of reference: the two plastic balls that were used. We choose the balls rather than the actual stems and fruits to avoid confusion from, e.g., varying width at different points along the stem. The larger ball had a diameter of 50 mm, which is similar to the size of a tomato, while the smaller ball had a diameter of 7.8 mm, similar to the width of a corn stem.

Given the distance of an object from the sensor, one can approximate its size or length. Given a two pixels in the image, and the depth data, one can compute their 3D coordinates [7]:

(1) $$\begin{array}{ccc} X & = & \frac{D_{x,y}\cdot \left(c_{x}-x\right)}{f_{x}}, \end{array}$$

(2) $$\begin{array}{ccc} Y & = & \frac{D_{x,y}\cdot \left(c_{y}-y\right)}{f_{y}}, \end{array}$$

(3) $$\begin{array}{ccc} Z & = & D_{x,y}, \end{array}$$

where $D_{x,y}$ is the distance to the pixel at coordinates x,y, $c_{x}$ and $c_{y}$ are the principal points and $f_{x}$ and $f_{y}$ are the focal lengths expressed in pixel units. We used $D_{x,y}$ as the average distance of two selected pixels.

Given the 3D coordinates for each pixel, measuring the distance between two pixels is simple, using Euclidean distance in 3D, denoted $d(p_{1},p_{2})$. Given a series of images, let $p_{1}^{i}$ and $p_{2}^{i}$ be two pixels on the opposite sides on the perimeter of the ball in image *i*. We can now compute the root of the mean square error (RMSE) of the estimation:

(4) $$RMSE=\sqrt{\frac{\sum_{i=1..N}{\left(d\left(p_{1}^{i},p_{2}^{i}\right)-\Phi \right)}^{2}}{N}},$$

where Φ is the true ball diameter. In some cases, it is interesting to compute relative error with respect to the size of the object. This is because an error of, e.g., 1 cm, may be problematic when estimating a distance of 10 cm, but may be considered negligible when estimating a distance of 1 m. We hence compute the mean relative average error (MRAE):

(5) $$MRAE=\frac{\sum_{i=1..N}\frac{{\left(d\left(p_{1}^{i},p_{2}^{i}\right)-\Phi \right)}^{2}}{\Phi }}{N}.$$

For estimating the ball diameter using the various sensors, we manually selected two pairs of pixels, positioned on two opposite sides of the ball perimeter. We then use Equations (1)–(3) to compute the 3D coordinates of the two pixels, and finally computed the Euclidean distance between a pair of points. We report the average of the two pairs as the computed ball diameter.

## 4. Results

The main goal of our study is to evaluate the usability of the various sensors for outdoor close range phenotyping. Below, we provide the results over the conducted experiment. We report the amount of the depth information that was captured, analyzing the fill rate of the sensors in varying conditions. We then report the quality of the RGB and the depth information that was captured for the important task of object identification. We then analyze the usability of the depth information for estimating object size, which is also often useful in phenotyping applications.

### 4.1. Fill Rate

**Figure 3:**
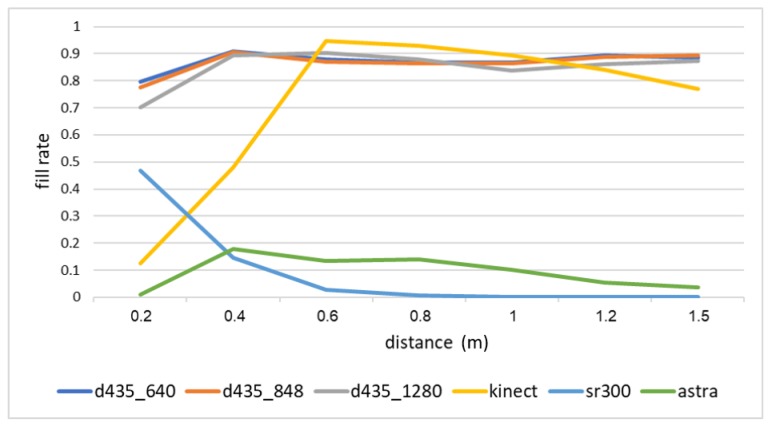
Mean fill rate over all objects of interest for each camera by distance to the objects.

Figure 3 shows the fill rate over all objects of interest by the distance to the objects. The Orbbec Astra s sensor produces the worst results here, with a fill rate of about 10% from 40 cm to 100 cm. The Intel sr300 depth sensor is designed only for short-range measurements. As such, it provides about 50% fill rate at 20 cm, 10% at 40 cm, and almost no depth information above that range. The Microsoft Kinect ii depth sensor is designed to operate in a range above 50 cm. Indeed, from 50 cm and on, the Microsoft Kinect ii sensor produces very good results, with about a 90% fill rate. Finally, the Intel d435 sensor, in all possible resolutions, provides the best overall performance, with the highest fill rate in short ranges (20 cm to 40 cm), and a comparable rate to Microsoft Kinect ii in the range of 60 cm to 150 cm. This is impressive, especially given the difference in power consumption between the two sensors.

We now analyze the sensitivity of the depth sensors to the lighting conditions. This is especially important in the uncontrolled field conditions that we are interested in, where lighting conditions may vary considerably. For this analysis, we consider only a distance range where the sensors operate well—above 40 cm for Microsoft Kinect ii, below 40 cm for Intel sr300, 40 cm to 100 cm for Orbbec Astra s, and all ranges for Intel d435.

**Figure 4:**
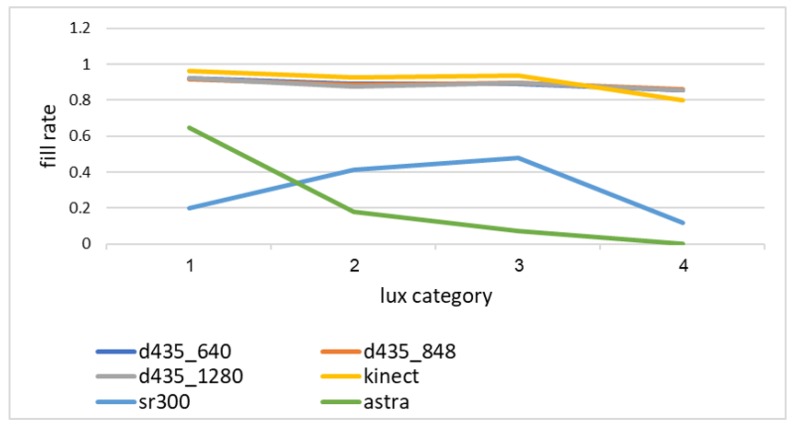
Fill rate by light intensity for all depth sensors, considering only objects within the camera range.Fill rate

Figure 4 shows the fill rate by light intensity. As can be seen, the Orbbec Astra s sensor operates best in the lowest lighting intensity measured, while the Intel sr300 sensor operates best in the medium lighting conditions, reaching a fill rate comparable to the best sensors at one specific time of day—early morning with medium lighting intensity. Both the Microsoft Kinect ii and the Intel d435 sensors showed little sensitivity to the lighting conditions, with a slight decrease in performance in the highest lighting conditions measured, around mid-day.

**Figure 5:**
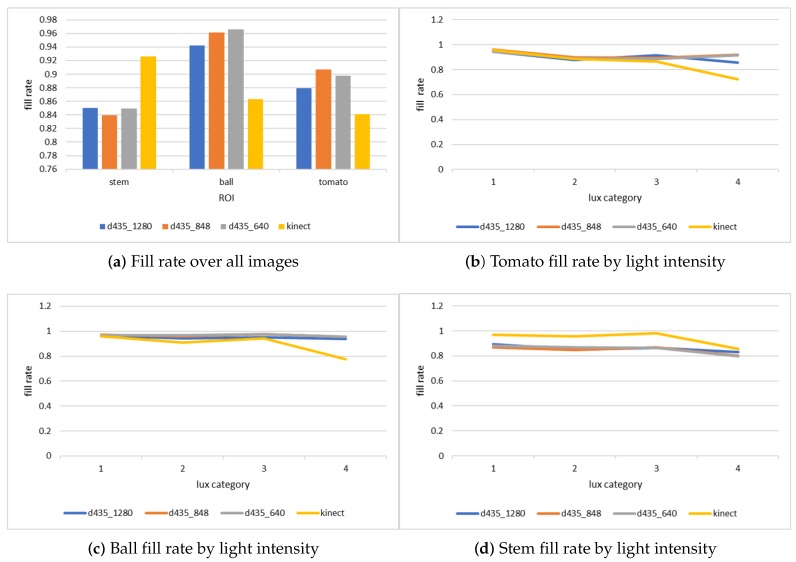
Comparing fill rate for different objects.

There are several factors that can affect the fill rate percentage such as an object’s shape and the reflection of light over the object. Figure 5 shows the fill rate for each object type, focusing on the two best sensors—Microsoft Kinect ii and Intel d435. The sensors vary in their behavior—the Intel d435 captures the ball best, has medium fill rate over the tomatoes, and the lowest performance on the stems, while the Microsoft Kinect ii has the best performance over the stems, and worse performance on both the tomatoes and the ball.

We performed statistical analysis to measure the significance of the differences between the sensors fill rate. We computed *p*-values for each object separately. We started with Levene’s test for homogeneity of variance in order to check the assumption of equal variances of fill rate for all cameras. For the three-objects type, the computed *p*-values were less than 0.05, i.e., the variances are not equal with high probability. We further performed a Welch one-way ANOVA test. The results for the three objects were significant with *p*-values under 0.001. For computing multiple pairwise comparison between the means of cameras, we used the Games–Howell post hoc test for unequal group size. The results are presented in Table 2. The test results show that most sensors are statistically different in their fill rate performances for each object. The Intel d435 variants with different resolutions were not significantly different, and the difference between the Microsoft Kinect ii and the Intel d435 sensor at the 1280 resolution is slightly above the 0.05 threshold.

When considering the light intensity, we see that the Intel d435 is unaffected by the light intensity in different hours for the ball, but suffers a slight reduction in quality for the stem fill rate. The Microsoft Kinect ii suffers a considerable reduction in quality during the hours when the sun light is strongest, but this reduction is less noticeable for the stems.

### 4.2. Object Detection Using Deep-Learning

First, we examine the performance of all RGB sensors in the detection task by training a model for each camera, using only the obtained RGB images. The train set contained 126 images, the validation set 14 images and the test set 28 images. Table 3 presents the mAP (mean average precision) results for each camera’s model. As the table shows, the Intel d435 RGB images produced the best results in the lowest resolution. The Orbbec Astra s RGB data produced the lowest quality object identification model. For statistical analysis, we used the sign test for matched pairs, when we say that one sensor is better than the other if it detects an object that the other missed. Despite the differences in mAP, the sign test did not found the differences to be significant. It may well be that, with more examples, statistical significance can be established.

We also experimented with using the depth information produced by the sensors to augment the RGB data in object identification [37]. As only the Microsoft Kinect ii and the Intel d435 sensors produced a reasonable fill rate for most ranges, we limit this experiment only to data gathered using these two cameras. We used only images captured within the camera’s working range, above 60 cm for Microsoft Kinect ii and above 40 cm for the Intel d435. Table 4 shows the image split into train, validation and test sets for each sensor.

We experimented with two methods for incorporating the depth information. First, we replaced the blue channel in the RGB image with the depth information. We hence trade some color information for the depth information. As can be seen in Table 5, this method helped in only a two cases—the Intel d435 sensor with the highest resolution and the Microsoft Kinect ii. For other Intel d435 resolutions, this method only reduced the performance. This may be due to insufficient training data, as the original Mask R-CNN network that we used was trained with standard RGB data, and may be incompatible with this type of data.

**Figure 6:**
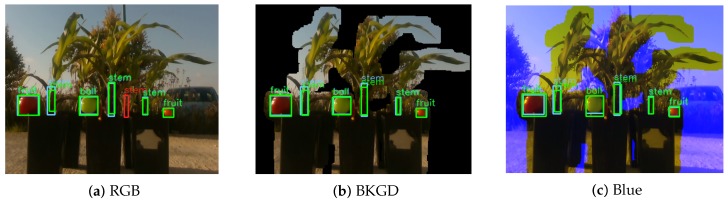
Intel d435:848 detection examples for each method. The blue bounding box denotes the ground truth, the green bounding box denotes a true positive prediction, and the red bounding box denotes a false positive prediction.

We next replaced the image background with black pixels. As we know the distance *d* between the camera to the object of interest, we only maintain the color of pixels within ±25 cm of *d*. To avoid removing pixels within the object of interest with missing or incorrect depth information, a pixel that had a neighbor within a 10 pixel radius that was in the ±25 cm range, was not modified. The color of all other pixels was changed to black. As can be seen in Table 5, this method improved object identification in all cases. Figure 6 provides an example of object identification using the three methods. As we can see, the removed background prevented the identification of a background object.

Performing the sign test, we cannot establish statistical significance of the differences in mAP.

### 4.3. Object Size Estimation

**Figure 7:**
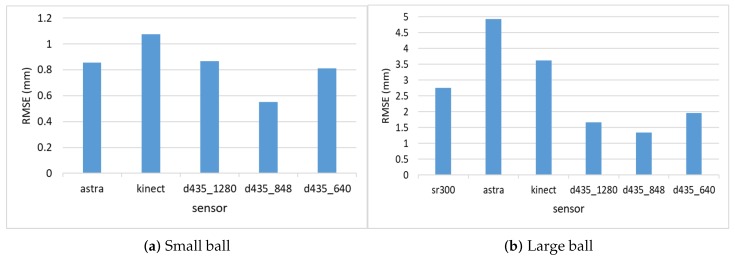
RMSE for diameter estimation of the two balls.

Figure 7 shows the RMSE results for estimating the diameter of the two balls. As can be seen, all Intel d435 resolutions performed better than all other sensors. For both balls, the 848 resolution provided the best depth information, and, hence, the best diameter estimation. The Orbbec Astra s sensor had the worst estimation with the highest error on the larger ball. This is because the Orbbec Astra s sensor did not capture well the distance to points on the perimeter of the larger ball from the perspective of the camera, possibly due to reflection angles. For the smaller ball, the Orbbec Astra s sensor provided much better estimations. The Intel sr300 sensor preformed well on the larger ball, but it is limited only to a close range, and was able to provide estimations only within a 40 cm distance, and was unable to capture the smaller ball at any range.

**Figure 8:**
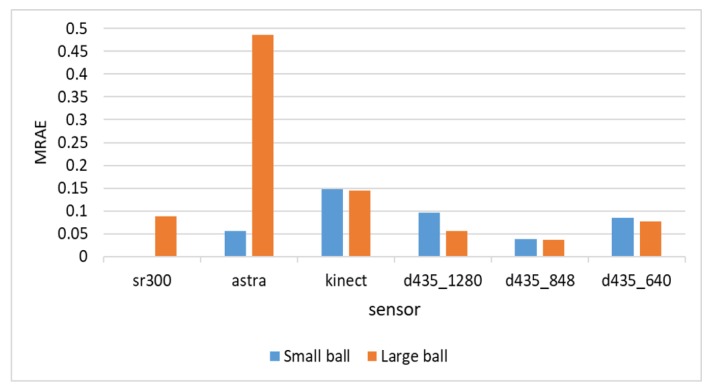
MRAE for diameter estimation of the two balls.

Figure 8 shows the relative error (MRAE) with respect to the ball diameter. We can see here that the Intel d435 sensor provided the best relative error, below 5% of the object diameter for the 848 resolution, and below 10% for the other resolutions.

**Figure 9:**
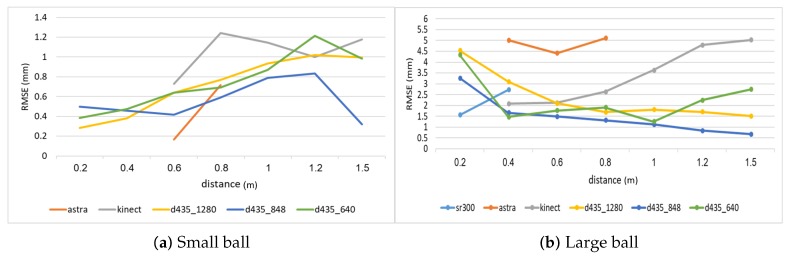
RMSE for diameter estimation of the two balls from varying distances.

Depth estimation often depends on the distance from the sensor to the object. Figure 9 shows the RMSE for varying object distances. The results are different for the various sensors. For the larger ball, the Microsoft Kinect ii sensor error grows with the distance to the target object, but the Intel d435 error is reduced with the distance, and, for the best resolution, 848, it is at its lowest at the 1.5 m range. The Intel sr300 works best at the 20 cm range, where it provides the best results, and reasonably well for the 40 cm range. The Orbbec Astra s sensor works poorly on all ranges here. For the smaller ball, the results are different, Intel d435 errors are not well correlated with the distance for the 848 resolution, while the error grows with the distance for the 1280 and the 640 resolution. For the smaller ball, the Orbbec Astra s sensor provides the best results at the 60 cm range, but much worse results for the 80 cm range. While on the larger ball the Orbbec Astra s collected depth information at 40 cm, for the smaller ball, the Orbbec Astra s could not capture depth information in the range of 40 cm.

**Figure 10:**
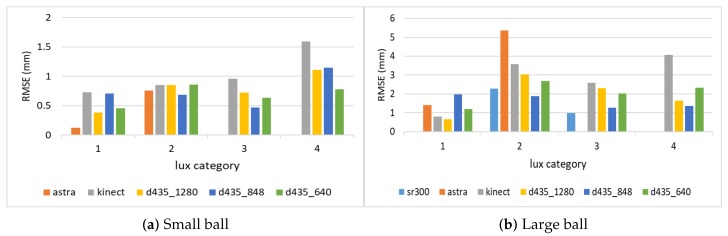
RMSE for diameter estimation of the two balls in varying light intensity categories.

Another factor that may affect distance estimation is the light intensity. Figure 10 shows the distance estimations in various lux categories. For the larger ball, the Intel d435 sensor is almost unaffected by light intensity. For the smaller ball, the Intel d435 estimation is worse as the light intensity grows. The Orbbec Astra s sensor performs the best at the lowest lux for the smaller ball, and is competitive at the lowest lux for the larger ball as well, but its performance is reduced substantially as the light intensity grows, failing to capture any meaningful depth information in the higher lux categories. The Microsoft Kinect ii sensor provides competitive results at lower light intensities, but its performance degrades at higher light intensities.

As we have seen above, the fill rate of the Intel sr300 and Orbbec Astra s are low. Hence, the amount of images containing sufficient depth information obtained by these two sensors (objects with a fill rate over 50%) is low compared with the Microsoft Kinect ii and the Intel d435 sensors. Moreover, Orbbec Astra s and Intel d435 are limited to shorter ranges. We will therefore separate analysis of the sensors.

To statistically analyze the differences between the sensors in squared error estimation, we used again Levene’s test for homogeneity of variance for both balls. For the large ball, we reject the null hypothesis with *p*-value < 0.001, i.e., we cannot assume the homogeneity of variances in the different sensors. For the small ball, the Levene’s *p*-value is 0.261, which is higher than the standard significance level of 0.05. That is, we cannot reject the homeginity of the variances for the smaller ball. For the larger ball, we further performed a Welch one-way test. The resulting *p*-value is less than 0.001, allowing us to conclude that the differences between the sensors are significant. The results of the Games–Howell test for multiple pairwise comparisons between the squared error means of the sensors are presented in Table 6. We can see that the Intel d435 variants have a significant difference in the large ball diameter estimation.

Finally, in many cases of agricultural applications, one is not necessarily interested in estimating the size of a specific object, but rather in the average size of objects of a given type in an area. For example, one may wish to compute the average tomato size in a greenhouse, or the average stem width in a corn field. In these cases, knowing the error distribution can help to compensate for errors in computing a better average estimation. Table 7 shows the distribution of errors for the Intel d435 and Microsoft Kinect ii sensors. As can be seen, the errors are in general Gaussian, and are often biased towards either positive or negative errors. In a future study, we will measure a large number of objects of a given type, and analyze different methods for taking the measurement noise into account.

## 5. Measuring Maize Stem Width Application

We now describe an application that we have developed for measuring the width of maize stems in field conditions, using the Intel d435 sensor that was shown to provide useful depth information in our experiments.

**Figure 11:**
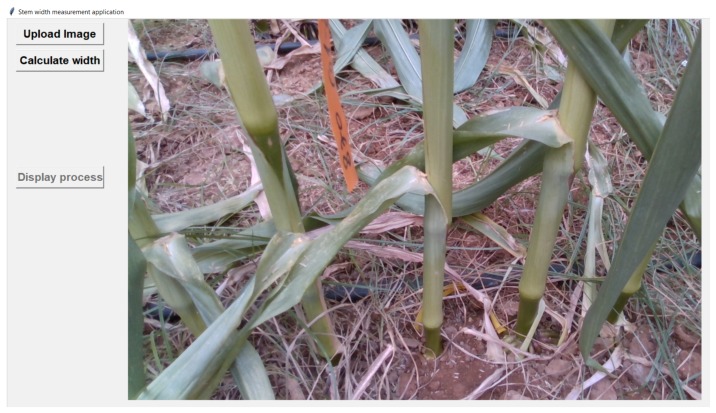
Screenshot of the maize stem width application. Showing an image of maize stems in a field, uploaded for width estimation.

Stem width is a key characteristic used for biomass potential evaluation of plants [38]. To estimate the width of maize stems, we developed an automated application, based on a deep-learning pipeline using the Intel d435 sensor images. The application allows users to upload RGB-D images from maize fields (Figure 11), for estimating the width of the detected stems in the image.

To build the deep learning models for our application, we used 100 RGB-D images, taken by the Intel d435, from 18 plots in a maize field. For all the training stages in our pipeline model, we used 80 images from 15 plots, and, for testing our model, we used 20 images from the three remaining plots.

**Figure 12:**
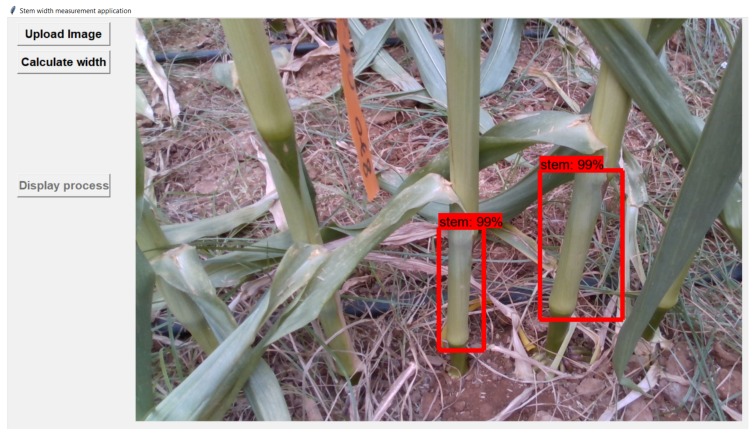
Detected stems in the image uploaded to the width estimation application.

Our application is based on two different models; first, we used tensorflow object detection API (https://github.com/tensorflow/models/tree/master/research/object_detection) for training a faster R-CNN model [39] for stem detection using the RGB images data only. Figure 12 shows the detected stems in the image. In addition, we trained a mask R-CNN model (https://github.com/matterport/Mask_RCNN) in order to perform segmentation on the stems that were detected by the first model.

**Figure 13:**
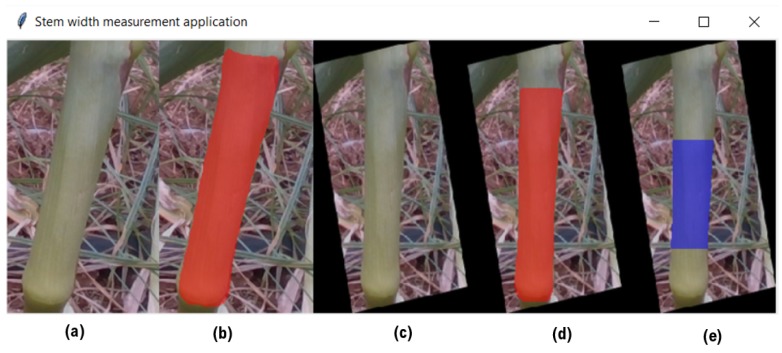
Visualization of stem width estimation process of the application. Red color mask indicates the segmentation result, and blue mask indicates the measured width lines. (**a**) We crop each of the detected stems from the RGB and depth images based on the identified bounding box coordinates. (**b**) We run the mask R-CNN model on the cropped RGB images of the detected stem. The segmentation for each stem is then used for estimating the orientation of the stem, used for canonization of the stems. The orientation estimation is done by finding the incline of the line between the highest and the lowest pixels that belong to the stem. (**c**) We rotate the cropped RGB and depth images according to the orientation angle in order to bring the stem into a vertical position. (**d**) We run the mask R-CNN again on the canonized RGB image of the stem in order to find the segmentation of the vertical stem. (**e**) We identify pixels in the mid-section of the stem, to be used for width estimation. The selected pixels are the leftmost pixels in the middle area of the segmentation and their corresponding rightmost pixels over the same horizontal lines. The algorithm ignores horizontal lines that their lengths are 3 standard deviations away from the average length of all the lines. The average distance, computed as in the ball diameter estimation above, of all unfiltered lines is reported as the stem width.

We begin with using the faster R-CNN model to detect the lower stems in the RGB images. We then use a pipeline algorithm with the following stages, demonstrated in Figure 13.

1. We crop each of the detected stems from the RGB and depth images based on the identified bounding box coordinates.
2. We run the mask R-CNN model on the cropped RGB images of the detected stem. The segmentation for each stem is then used for estimating the orientation of the stem, used for canonization of the stems. The orientation estimation is done by finding the incline of the line between the highest and the lowest pixels that belong to the stem.
3. We rotate the cropped RGB and depth images according to the orientation angle in order to bring the stem into a vertical position.
4. We run the mask R-CNN again on the canonized RGB image of the stem in order to find the segmentation of the vertical stem.
5. We identify pixels in the mid-section of the stem, to be used for width estimation. The selected pixels are the leftmost pixels in the middle area of the segmentation and their corresponding rightmost pixels over the same horizontal lines. The algorithm ignores horizontal lines that their lengths are three standard deviations away from the average length of all the lines. The average distance, computed as in the ball diameter estimation above, of all unfiltered lines is reported as the stem width.

**Figure 14:**
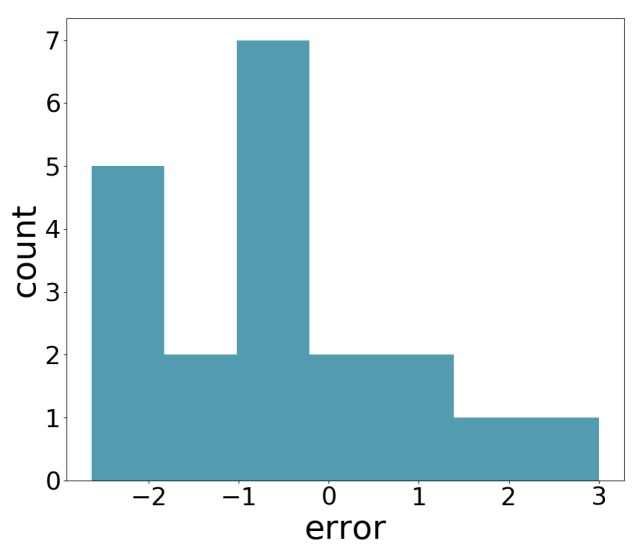
Error distribution.

We compared the application estimation results on 20 stems from the tested three plots, with manual measurements done by caliper. The RMSE results are 1.58 mm. In Figure 14, we can see the error distribution for the 20 stem measurements.

Of course, the evaluation of our application requires more rigorous testing with much larger train and test sets, and we report here only preliminary proof of concept results. That being said, the developed application demonstrates the potential of RGB-D sensors for phenotyping applications.

## 6. Discussion

All the analysis provided in the previous section clearly shows that the new Intel d435 sensor can be very useful in field agricultural applications. The Intel d435 produces RGB data of sufficient quality for object identification using image-based deep learning methods, which is an important task in agriculture. The depth information produced by the sensor also has the highest quality of the sensors that we experiment with. First, the Intel d435 is able to produce a competitive fill rate over all the ranges in our experiments—from 20 cm to 1.5 m. Furthermore, when using the depth measurements for object size estimation, which is also an important task in agriculture, the Intel d435 sensor provided the best results.

Developing phenotyping tools and applications which are operational automated and accurate can be challenging in the presence of occlusions. Steinhage et al. [40] and Nguyen et al. [41] deal with the occlusion challenge by developing 3d reconstruction models using laser scanning or multiply cameras, which are high cost sensors. Additional research is needed to understand whether the data information computed by the low cost RGB-D sensors that we study can help in identifying and resolving occlusions. That being said, it might be that, for statistical applications, that attempt to compute a phenotype, such as the maize stem width, in an entire field, one can use only non-occluded objects (stems), to achieve a reliable average over all the field. If objects are occluded at random, one should expect the non-occluded objects to be a good sample of all the objects. In other applications, such as watermelons, where most fruits are hidden by leaves, it is unlikely that simple RGB-D sensors will supply sufficient information for computing, e.g., the average fruit diameter.

The Intel d435 sensor supports three different resolutions. In our experiments, the 848 resolution provided the best depth information, resulting in lower errors in object size estimation. The RGB information of the lower 640 resolution provided better object identification capabilities. The better performance for object identification may be attributed to the properties of the deep learning algorithm, rather than the more accurate RGB data.

The Microsoft Kinect ii sensor also provides a competitive fill rate at all ranges, but the depth measurement quality is lower at the larger distances, and also at higher light intensities. In addition, the Microsoft Kinect ii has substantially higher energy requirements than the other sensors, which may pose a problem in applications that require a low weight sensor, such as in small drone applications.

The Intel sr300 sensor provides competitive results at lower ranges, especially for measuring larger objects sizes, but it is limited only for close-range applications.

Finally, the Orbbec Astra s sensor did not perform well in our experiments, and may not be the best choice for outdoor applications.

## 7. Conclusions

In this research, we experiment with four RGB-D sensors—Microsoft Kinect ii, Orbbec Astra s, Intel sr300, and Intel d435 with three different resolutions—in outdoor agricultural-oriented tasks. We compute the fill rate of the sensors over various agricultural objects, and estimate the quality of the depth information for measuring object size. We also use both the RGB and the depth data captured by the sensors for object identification using deep learning methods.

Our experiments clearly show that the new Intel d435 sensor provides the best results in our settings, and is hence a viable alternative for outdoor agricultural applications. The relatively high quality depth information, together with low energy requirements, and low weight, make the Intel d435 useful for many field applications, such as drone-based phenotyping tasks.

In the future, we intend to use the Intel d435 in field phenotyping tasks, similar to our application for measuring the average diameter of stems in maize. We intend to extend our application to handle stems of wheat and tomatoes in the field, and also to estimate the average fruit size in various crops. We also intend to mount the Intel d435 on a drone to test its applicability for drone-based phenotyping applications. Using RGB-D sensors for the tasks above in fields conditions will allow us to examine the value of the depth information for handling field-based phenotyping challenges such as occlusion.

## Figures and Tables

**Table 1:** Properties of the RGB-D sensors used in our experiment.

	Sensor			
**Parameter**	**Orbbec Astra s**	**Microsoft Kinect ii**	**Intel sr300**	**Intel d435**
Range	0.4 m–2 m	0.5 m–4.5 m	0.3 m–2 m	0.2 m–10 m
RGB FOV	60${}^{\circ }$(H) × 49.5${}^{\circ }$(V) × 73${}^{\circ }$(D)	70.6${}^{\circ }$(H) × 60${}^{\circ }$(V)	41.5${}^{\circ }$(H) × 68${}^{\circ }$(V) × 75.2${}^{\circ }$(D)	69.4${}^{\circ }$(H) × 42.5${}^{\circ }$(V) ) × 77${}^{\circ }$(D)
Depth FOV	60${}^{\circ }$(H) × 49.5${}^{\circ }$(V) × 73${}^{\circ }$(D)	70.6${}^{\circ }$(H) × 60${}^{\circ }$(V)	55${}^{\circ }$(H) × 71.55${}^{\circ }$(V) × 88${}^{\circ }$(D)	91.2${}^{\circ }$(H) × 65.5${}^{\circ }$(V)) × 100.6${}^{\circ }$(D)
Frame rate	30 fps	30 fps	30, 60 fps	30, 60, 90 fps
RGB resolution	640 × 480 pixel	1920 × 1080 pixel	1920 × 1080 pixel	1920 × 1080 pixel
				1280 × 720 pixel
				848 × 480 pixel
				640 × 480 pixel
Depth resolution	640 × 480 pixel	512 × 424 pixel	640 × 480 pixel	1280 × 720 pixel
				848 × 480 pixel
				640 × 480 pixel
Weight	300 g	966 g	300 g	100 g
Size	165 mm × 30 mm × 40 mm	255 mm × 66 mm × 67 mm	110 mm × 12.6 mm × 4.1 mm	90 mm × 25 mm × 25 mm
Power supply	USB 2.0	power supply + USB 3.0	USB 3.0	USB 3.0
Power consumption	<2.4 W	∼15 W	650–1800 mW	618–1978 mW
Operating	Android, Linux	Linux	Linux	Linux
system	Windows 7/8/10	Windows 8/10	Windows 8/10	Window 8/10
SDK	Astra SDK	Kinect V2 SDK	Intel©RealSense^TM^	Intel©RealSense^TM^ SDK
	OpenNI2	libfreenect2	SDK	librealsense SDK ${}^{1}$
	3rd party S		librealsense sdk ${}^{2}$	hand and face tracking

${}^{1}$https://github.com/IntelRealSense/librealsense; ${}^{2}$https://github.com/IntelRealSense/librealsense. (H)—Horizontal, (V)—Vertical, (D)—Diagonal.

**Table 2:** Games–Howell test results for tomato, ball and stem. Bolded results are significant at the 0.05 level.

	Tomato			Ball			Stem		
Sensor A–Sensor B	t	df	*p*-Value	t	df	*p*-value	t	df	*p*-Value
sr300–astra	4.879	78.263	**<0.001**	1.836	64.355	0.450	3.731	206.825	**0.003**
sr300–d435:640	16.703	67.034	**<0.001**	11.201	39.219	**<0.001**	18.154	131.365	**<0.001**
sr300–kinect	14.852	73.432	**<0.001**	9.211	42.772	**<0.001**	21.089	121.336	**<0.001**
sr300–d435:1280	16.321	65.258	**<0.001**	10.783	39.383	**<0.001**	18.205	130.635	**<0.001**
sr300–d435:848	17.058	65.296	**<0.001**	11.116	39.207	**<0.001**	17.734	134.278	**<0.001**
astra–d435:640	51.847	286.481	**<0.001**	23.828	96.722	**<0.001**	33.019	384.376	**<0.001**
astra:kinect	42.828	357.485	**<0.001**	19.289	124.100	**<0.001**	38.414	327.718	**<0.001**
astra–d435:1280	52.728	254.347	**<0.001**	23.022	98.019	**<0.001**	33.159	380.361	**<0.001**
astra–d435:848	54.498	255.086	**<0.001**	23.680	96.630	**<0.001**	32.154	400.064	**<0.001**
d435:640–kinect	4.004	403.167	**0.001**	7.965	130.797	**<0.001**	7.138	700.288	**<0.001**
d435:640–d435:1280	1.750	493.051	0.499	4.634	266.150	**<0.001**	0.063	803.813	0.999
d435:640–d435:848	0.860	493.771	0.956	1.161	285.786	0.855	0.764	804.866	0.973
kinect–d435:1280	2.829	360.864	0.055	6.029	141.119	**<0.001**	7.149	702.384	**<0.001**
kinect–d435:848	4.885	361.930	**<0.001**	7.594	130.061	**<0.001**	7.701	684.084	**<0.001**
d435:1280–d435:848	2.860	509.990	**0.050**	3.693	262.636	**0.004**	0.831	801.180	0.962

**Table 3:** mAP results for the object detection task using only RGB data.

Sensor	mAP	Tomato	Ball	Stem
Intel d435:640	0.955	1	0.99	0.87
Intel d435:1280	0.906	1	1	0.72
Intel sr300	0.925	1	0.95	0.83
Intel d435:848	0.912	1	1	0.74
Microsoft Kinect ii	0.914	1	1	0.75
Orbbec Astra s	0.826	0.96	0.88	0.64

**Table 4:** Train, validation, and test splits for depth integration in object detection.

Set	Microsoft Kinect ii	Intel d435
Train	86	112
Validation	8	10
Test	18	22

**Table 5:** mAP results for object identification incorporating depth information for all object types. RGB denotes using only the RGB data, BKGD denotes background removal prior to identificaiton, and Blue denotes replacing the blue channel with depth information.

	All			Tomato			Ball			Stem		
Sensor	RGB	BKGD	Blue	RGB	BKGD	Blue	RGB	BKGD	Blue	RGB	BKGD	Blue
Kinect	0.866	0.924	0.9	1	1	1	0.9	0.96	0.9	0.7	0.81	0.81
D435:1280	0.912	0.932	0.938	0.97	0.97	0.98	0.92	0.95	0.92	0.85	0.87	0.92
D435:848	0.962	0.971	0.936	1	1	1	1	1	1	0.89	0.91	0.81
D435:640	0.976	0.981	0.953	1	1	1	1	1	1	0.93	0.94	0.86

**Table 6:** Games–Howell test results for large ball size estimation. Significant results are bolded.

Sensor Pair	t	df	*p*-Value
sr300–astra	6.146	43.997	**<0.001**
sr300–d435:640	0.331	39.734	0.999
sr300–kinect	5.576	120.889	**<0.001**
sr300–d435:1280	1.244	40.900	0.813
sr300–d435:848	2.179	34.336	0.274
astra–d435:640	6.621	35.905	**<0.001**
astra–kinect	3.397	45.408	**0.017**
astra–d435:1280	6.962	36.056	**<0.001**
astra–d435:848	7.325	35.185	**<0.001**
d435:640–kinect	7.742	261.746	**<0.001**
d435:640–d435:1280	2.099	652.141	0.289
d435:640–d435:848	5.399	466.849	**<0.001**
kinect–d435:1280	8.577	268.453	**<0.001**
kinect–d435:848	9.787	229.301	**<0.001**
d435:1280–d435:848	2.423	433.703	0.151

**Table 7:** Error distribution.

Ball	Kinect	D435:640	D435:848	D435:1280
Large	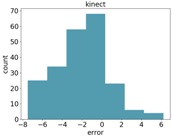	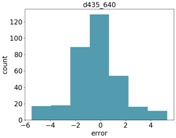	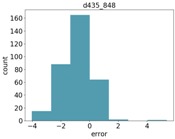	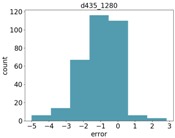
Small